# A Tunable Photoluminescent Composite of Cellulose Nanofibrils and CdS Quantum Dots

**DOI:** 10.3390/nano6090164

**Published:** 2016-09-07

**Authors:** Qinwen Wang, Aimin Tang, Yuan Liu, Zhiqiang Fang, Shiyu Fu

**Affiliations:** State Key Laboratory of Pulp and Paper Engineering, South China University of Technology, Guangzhou 510640, China; qwwang@scut.edu.cn (Q.W.); l.yuan27@mail.scut.edu.cn (Y.L.); fangzq1230@126.com (Z.F.); shyfu@scut.edu.cn (S.F.)

**Keywords:** cellulose nanofibrils, CdS, quantum size effect, photoluminescence performance

## Abstract

The preparation of fluorescent nanocomposite materials with tunable emission wavelengths by combining cellulose nanofibrils (CNFs) with inorganic nanoparticles is important for promoting CNFs applications. A CNF/CdS nanocomposite was prepared via in situ compositing at room temperature on oxidized CNFs with CdS quantum dots. By controlling the –COOH/Cd^2+^ ratio on the CNF, the feeding time of Na_2_S and the ultrasonic maturing time, the size of the CdS quantum dots on the CNF surface could be adjusted so that to obtain the CNF/CdS nanocomposite material with different fluorescent colors. The results indicated that the CdS particles quantized were evenly distributed on the CNF. The maximum average size of the CdS nanoparticles glowed red under the excitation of UV light was 5.34 nm, which could be obtained with a –COOH/Cd^2+^ ratio of 1.0, a Na_2_S feeding time of 20 min, and an ultrasonic maturing time of 60 min. A series of CNF/CdS nanocomposite materials were obtained with CdS nanoparticle sizes varying from 3.44 nm to 5.34 nm, the emission wavelength of which varied from 546 nm to 655 nm, and their fluorescence color changed from green to yellow to red. This is the first time the fluorescence-tunable effect of the CNF/CdS nanocomposite has been realized.

## 1. Introduction

CdS is a typical II–VI semiconductor and its particles exhibit good optical and photocatalytic properties, and tunable photoluminescence [1]. The different photoluminescence performances of CdS nanoparticles have different potential applications in fields such as biomarker [2], photoelectric devices [3], sensors [4], and ion detection [5]. Researchers have found that the optical properties of CdS nanoparticles can be adjusted by controlling their size, size distribution and surface coating. For example, Pattabi et al. [6] studied the preparation and stability of thiophenol-capped CdS nanoparticles. They found that the size of the CdS nanoparticles could be changed by adjusting the concentration of the thiophenol stabilizer. With an increase in stabilizer concentration, the obtained CdS particle sizes decreased. The optical absorption edge showed a blue shift, thereby realizing the tunable fluorescence. Chen et al. [7] prepared CdS/ZnS core/shell nanocrystals with a single-source precursor. The fluorescence characteristics of the CdS nanocrystals were adjusted by controlling the growth layers of the ZnS shell. It was found that the emission peak of the nanocrystals showed a gradual redshift with an increasing number of ZnS shell growth layers.

However, CdS nanoparticles are easily aggregated due to their high surface free energy. Therefore, control of the particle size and stability is a key issue that needs to be solved. At present, inorganic-organic composite materials exhibit better mechanical, optical and electrical properties and thermal stability than those of single materials, and they have attracted significant attention in many areas, including electrical equipment, solar devices, coatings, fuel cells, and sensors [8]. Petrochemical polymer products, including polyvinylpyrrolidone [9], poly(ethylene glycol)-*block*-poly(ethyleneimine) [10], and perfluorinated sulfonic acid [11], can be used for compositing with CdS particles. However, for environmental reasons, the existing petrochemical products are being gradually replaced by bio-based polymer materials. Silk protein fiber [12] and bacterial cellulose [1] have advantages in environmental protection and degradability, and have also been used for the preparation of CdS composites.

Due to their advantages, such as renewability, degradability, large specific surface area, and a high number of active groups, plant-based cellulose nanofibrils (CNFs) have been widely used in many fields, including flexible electronic devices, cosmetics, and food additives. There have been numerous reports [13,14,15,16] that the surface-active groups of CNFs can adsorb cations such as Mn, Cd, Fe, and Zn. This characteristic is very advantageous for the control of compositing sites of inorganic nanoparticles, as well as the preparation of CNF/inorganic nanocomposites with uniform particle size distributions, which is realized through the adsorption of active groups and precursor ions by an in-situ compositing method. In a previous study, Li et al. [1] prepared a bacterial cellulose/CdS nanocomposite material. They believed that the hydroxyl groups in cellulose were reaction sites that could be used to control the distribution of CdS. In addition, the photoluminescence performance was also investigated. It was found that the ultraviolet absorption and emission peaks of the bacterial cellulose/CdS nanocomposite material showed a blue shift compared to the CdS bulk material. The plant-based CNFs prepared by TEMPO oxidization and mechanical treatment have carboxyl groups (–COOH). However, the questions of whether these carboxyl groups have the same functions as hydroxyl groups and how to prepare fluorescence tunable plant-based CNF/CdS nanocomposites have not yet been answered. In this study, CdS quantum dots were composited on CNF via in-situ compositing to prepare a CNF/CdS nanocomposite material. The CdS particle size in the CNF/CdS nanocomposite material was adjusted by varying the ratio of active groups (–COOH) on the CNF surface and Cd^2+^, the feeding time of Na_2_S and the ultrasonic maturing time. Consequently, the photoluminescence performances of the obtained material were adjustable. These results provide a basis for the preparation of emission wavelength tunable CNFs/CdS nanocomposite materials, which are applicable for many applications, including biomarkers, photoelectric devices, sensors, and ion detection.

## 2. Results and Discussion

In the preparation process of CNF/CdS nanocomposite via in situ compositing, a number of factors may have influences on the CdS particles size and size distribution and, thus, the photoluminescence performance of the CNF/CdS nanocomposite may change. The involved factors include the adsorption of Cd^2+^ precursor on CNFs, the generation of CdS and its compositing on the CNFs. This paper mainly studies the following three aspects, the -COOH/Cd^2+^ ratios, the Na_2_S feeding time and the ultrasonic maturing time.

### 2.1. Effect of –COOH/Cd^2+^ Ratio

**Figure 1:**
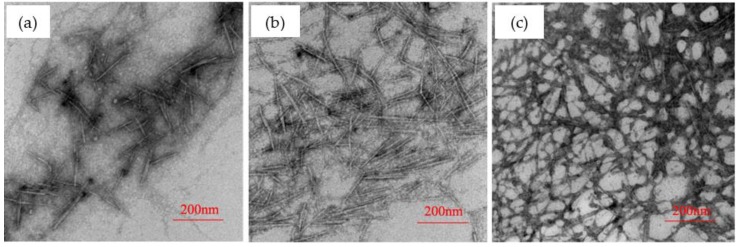
The TEM images of CNF/CdS nanocomposites with different –COOH/CdS ratios; (**a**) –COOH/Cd^2+^ = 0.5; (**b**) –COOH/Cd^2+^ = 1.0; and (**c**) –COOH/Cd^2+^ = 2.0 (the feeding time of Na_2_S was 20 min, and the ultrasonic maturing time was 60 min).

**Figure 2:**
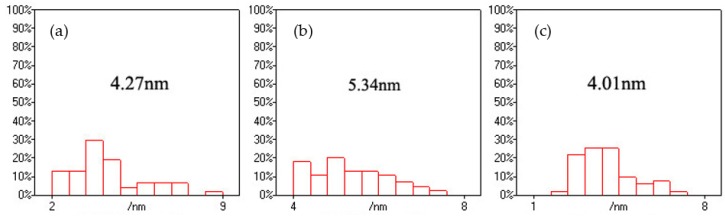
The size distributions of the CdS nanoparticles with different –COOH/CdS ratios; (**a**) –COOH/Cd^2+^ = 0.5; (**b**) –COOH/Cd^2+^ = 1.0; and (**c**) –COOH/Cd^2+^ = 2.0.

Firstly, the effects of –COOH/Cd^2+^ ratios on the morphology structure of the CNFs/CdS nanocomposite material were studied. The experiment was carried out on the following conditions: the feeding time of Na_2_S was 20 min, the ultrasonic maturing time was 60 min, and the –COOH/Cd^2+^ ratios were 0.5, 1.0, and 2.0. The results are shown in Figure 1. From Figure 1, the particle size and distribution of CdS were obtained using Nano Measurer software (Microsoft Corporation, Redmond, WA, USA). The results are shown in Figure 2.

Figure 1 presents the distribution change of CdS nanoparticles on the CNFs with the variation of the –COOH/Cd^2+^ ratio. The black points are CdS nanoparticles. It could be seen from the transmission electron microscope (TEM) image that the CdS nanoparticles were all uniformly dispersed onto the CNFs for three CNF/CdS nanocomposites prepared with different –COOH/Cd^2+^ ratios. This indicated that the CdS nanocomposite materials with CNFs as organic carriers could be successfully prepared by an in situ compositing method. The generation position of CdS was controlled by the position of –COOH on the CNFs.

Figure 2 shows the size distribution of the CdS nanoparticles. When the –COOH/Cd^2+^ ratio was 0.5, the average size of CdS nanoparticles in the CNF/CdS nanocomposite was 4.27 nm, and the particle size was distributed in the range of 2–9 nm. The particles with sizes of 3.3–4.7 nm accounted for 50% of all CdS particles, indicating the wide size distribution. In other words, the uniformity of CdS particle size was poor. When the –COOH/Cd^2+^ ratio was 1.0, the average size of CdS was clearly increased to 5.34 nm. Moreover, the particle sizes were fully distributed in the range of 4.0–7.5 nm. The distribution width was lower than that of the sample with a –COOH/Cd^2+^ ratio of 0.5. Therefore, the uniformity of CdS particle size distribution was improved. When the –COOH/Cd^2+^ ratio was 2.0, the average particle size of CdS was reduced to 4.01 nm. In this case, the particles with sizes of 2.3–4.3 nm accounted for 72% of particles, indicating the further improvement in the uniformity of particle size distribution. Dai et al. [17] found that the size of CdS nanoparticles also increases with increasing precursor (CdCl_2_ and Na_2_S) concentration. In this study, varying the ratio of –COOH and Cd^2+^ was used to control the adsorption amount of Cd^2+^ onto the CNFs. As a result, the size of the generated CdS nanoparticles was adjusted. Moreover, the distribution and distribution uniformity of the CdS nanoparticles on the CNFs were controlled through the localized adsorption of Cd^2+^ by –COOH. When the –COOH/Cd^2+^ ratio was 0.5, the amount of Cd^2+^ adsorbed by the CNFs was small. Therefore, the average size of CdS nanoparticles in the CNF/CdS nanocomposite was small. The compositing amount was also relatively low. When the –COOH/Cd^2+^ ratio was increased to 1.0, the content of –COOH increased, leading to an increase in the amount of Cd^2+^ adsorbed by the CNFs. After adding Na_2_S, the number of generated CdS nanoparticles increased. Meanwhile, the particle size also increased. When the –COOH/Cd^2+^ ratio was 2.0, the content of –COOH on the CNFs further increased; thus, the numbers of composited CdS nanoparticles increased. In addition, due to the chemical anchoring effect of –COOH on CNFs, the adsorbed Cd^2+^ was uniformly distributed onto the CNFs. Therefore, the number of generated CdS crystal nuclei increased. The particle size of CdS decreased while the distribution uniformity improved.

**Figure 3:**
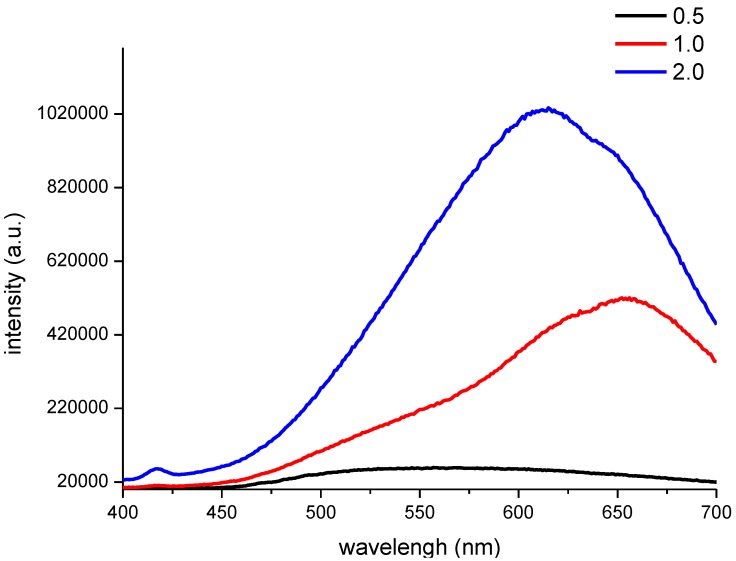
The PL spectra of the CNF/CdS nanocomposites with different –COOH/Cd^2+^ ratios (excitation wavelength 365 nm).

Furthermore, the PL spectra of the CNF/CdS nanocomposites with different –COOH/Cd^2+^ ratios were investigated. As shown in Figure 3, at an excitation wavelength of 365 nm, a strong emission peak with an intensity of 420,000 a.u. was observed at 655 nm for the CNF/CdS nanocomposite material when the –COOH/Cd^2+^ ratio was 1.0. The emission peak blueshifted to 613 nm when the –COOH/Cd^2+^ ratio was 2.0. However, the fluorescence intensity was at a maximum (1,020,000 a.u) at this time. When the –COOH/Cd^2+^ ratio was 0.5, the CNF/CdS nanocomposite had a very wide emission bandwidth at 500–625 nm, and the intensity was only 20,000 a.u. By comparing the data in Figure 2, it could be found that the CdS particle size distribution range was wide, the size and number of composited CdS particles was small when the –COOH/Cd^2+^ ratio was 0.5. As a result, the resulting CNF/CdS nanocomposite exhibited a wide fluorescence emission band with low intensity. When the –COOH/Cd^2+^ ratio increased, the size distribution uniformity of the CdS particles was gradually improved. Meanwhile, the number of composited CdS nanoparticles increased. Therefore, the fluorescence emission band of the CNF/CdS nanocomposite was gradually narrowed and the intensity was enhanced significantly. In addition, it has been reported [18] that a redshift phenomenon occurs with increasing CdS particle size, as a result of the quantum size effect. When the –COOH/Cd^2+^ ratio was decreased from 2.0 to 1.0, the average size of the CdS nanoparticles composited onto the CNFs increased from 4.01 nm to 5.34 nm, so that the PL spectra of the material showed the redshift phenomenon. Therefore, in this study, the PL spectra of the CNF/CdS nanocomposite were consistent with the size distribution results of CdS nanoparticles.

### 2.2. Effect of Na_2_S Feeding Time

**Figure 4:**
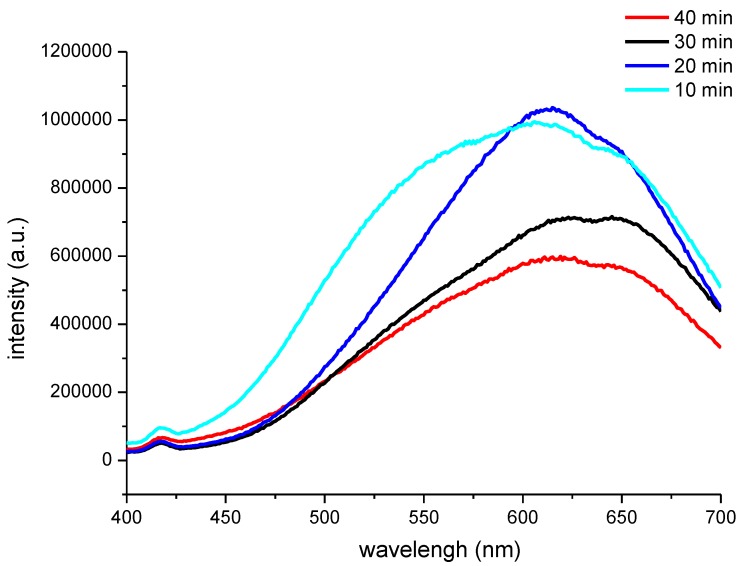
The PL spectra of the CNF/CdS nanocomposites with different Na_2_S feeding time (excitation wavelength 365 nm).

In the condition of that the CNFs carboxyl contents was 1.63 mmol/g, –COOH/Cd^2+^ ratio was controlled at 2.0 and ultrasonic maturing time was fixed at 60 min, the effect of Na_2_S feeding time on the PL spectrum of the CNF/CdS nanocomposite material was investigated. The results are shown in Figure 4.

It could be seen from Figure 4 that the PL peak of the CNF/CdS nanocomposite was 605 nm (excitation wavelength, 365 nm) when the feeding time of Na_2_S solution was 10 min. Moreover, the emission band appeared in the range of 500–650 nm with a serious broadening phenomenon. When the feeding time of Na_2_S solution was prolonged to 20 min and 30 min, the PL peaks showed redshifts at 613 nm and 640 nm, respectively. If the feeding time of Na_2_S solution was further prolonged to 40 min, the PL peak at 625 nm showed a blue shift. It was generally accepted that the high energy luminescence band (300–500 nm) of CdS was due to the band gap, while the low energy luminescence band (500–700 nm) was related to the surface defects. The wavelengths of the PL peaks of the CNF/CdS nanocomposite in this study were all higher than 600 nm, indicating that they were mainly due to the surface defects emission.

**Figure 5:**
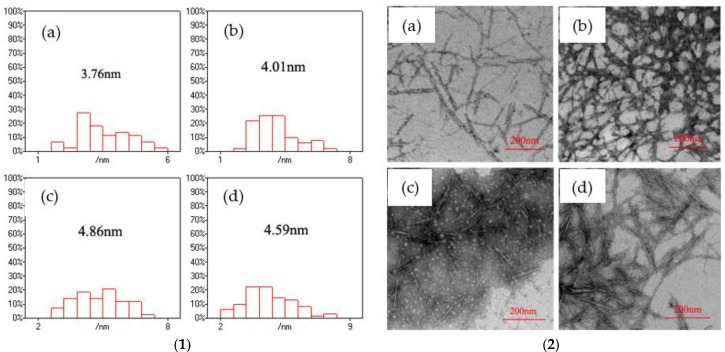
(**1**) The size distributions of the CdS nanoparticles with different Na_2_S feeding time; and (**2**) the TEM images of CNF/CdS nanocomposites with different Na_2_S feeding time; (**a**) 10 min; (**b**) 20 min; (**c**) 30 min; and (**d**) 40 min (–COOH/Cd^2+^ ratio 2.0, ultrasonic maturing time 60 min).

In order to reveal the reasons why the CNF/CdS nanocomposites prepared with different Na_2_S feeding time shows different luminescence, TEM analysis and Nano Measurer software was used to statistically calculate the CdS particle sizes. Figure 5 presents the size distribution of CdS nanoparticles, which was obtained using the Nano Measurer software based on a TEM image of the CNF/CdS nanocomposite. It indicated that the CdS particle sizes in the CNF/CdS nanocomposite were 3.76 nm and 4.01 nm when the feeding time of Na_2_S was 10 min and 20 min, respectively. The average size of the CdS nanoparticles increased to 4.86 nm when the feeding time of Na_2_S was 30 min. With the further prolonging of the Na_2_S feeding time to 40 min, the CdS particle size decreased to 4.59 nm. This could be explained by the fact that when the feeding time of Na_2_S was less than 20 min (relatively fast dropping speed), the number of generated CdS crystal nuclei was large. However, the crystalline grains were not fully grown within a short time, so the particle size was small. When the feeding time was prolonged to 30 min, the formation reaction of CdS nanoparticles was basically complete, and the average size of the obtained CdS nanoparticles was at a maximum. If the feeding time of Na_2_S was further increased (relatively slow dropping speed), there was not enough S^2−^ during the formation reaction of CdS. As a result, the average size of the CdS nanoparticles decreased. The studies of Martinez-Castanon et al. [19] showed the S^2^^−^:Cd^2+^ molar ratios affected the growth and size of particles. It was observed that the particle size increased with the S^2^^−^ concentration in precursors during the reaction. The different feeding time of Na_2_S made the different S^2^^−^:Cd^2+^ molar ratios in unit time.

The work of Pattabi et al. [20] indicated that the absorption spectrum of CdS nanoparticles showed a blueshift when particle size decreased. As the particle size decreased, the blueshift increased. This was associated with the quantum confinement effect. According to [21], the band gap of a CdS quantum dot is in accordance with the Brus equation (Equation (1)) of the absorption band edge movement:

(1) $$E_{1}=E_{g}+\frac{h^{2}\pi^{2}}{2m^{*}R^{2}}-\frac{1.8e^{2}}{\varepsilon R}-0.25E_{\mathrm{Ry}},$$

where $m^{*}={\left(\frac{1}{m_{e}}+\frac{1}{m_{h}}\right)}^{-1}$, where *E*_1_ is the band gap energy of the quantum dot, *R* is the radius of the quantum dot, *m_e_* and *m_h_* are the effective mass of an excited electron and excited hole, respectively, *E_g_* is the band gap energy of the bulk semiconductor, *E*_Ry_ is a parameter related to the exciton binding energy of the bulk semiconductor, and h is Planck’s constant.

According to the Brus equation, when the radius of quantum dot (*R*) is small enough, the band gap will increase with decreasing nanoparticle size, resulting in the blue shift of absorption peaks. It could be seen from Figure 4 and Figure 5 that the average size of the CdS nanoparticles was 4.86 nm when the feeding time of Na_2_S was 30 min, and the PL peak of the CNF/CdS nanocomposite was 640 nm. When the feeding time of Na_2_S was 40 min, 20 min, and 10 min, the average sizes of the CdS nanoparticles decreased to 4.59 nm, 4.01 nm and 3.76 nm, respectively. Accordingly, the PL peaks of the CNF/CdS nanocomposite showed blue shifts at 625 nm, 613 nm and 605 nm, respectively. Therefore, the PL peaks of the CNF/CdS nanocomposite were in the range of 640–605 nm, with only a small difference occurring when the feeding time of Na_2_S was changed. These results indicated that the size of the CdS nanoparticles could be changed by varying the feeding time of Na_2_S in order to adjust the PL spectrum of the CNF/CdS nanocomposite. However, the adjustable degree was not large.

### 2.3. Effect of Ultrasonic Maturing Time

**Figure 6:**
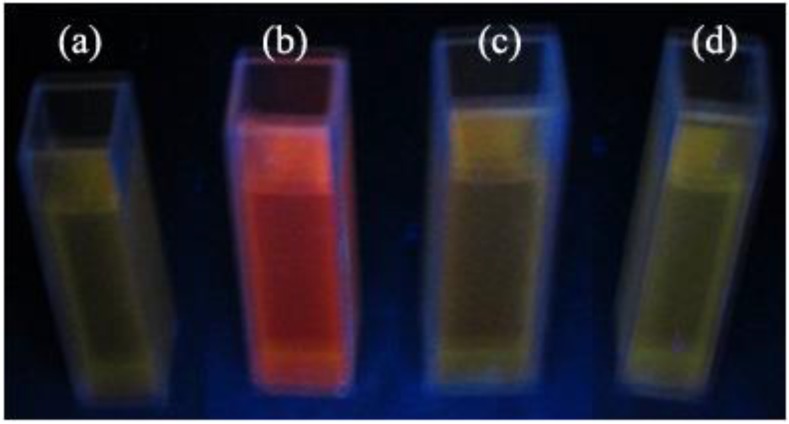
The fluorescence images of the CNF/CdS nanocomposites under different maturing time; (**a**) 40 min; (**b**) 60 min; (**c**) 80 min; and (**d**) 100 min.

**Figure 7:**
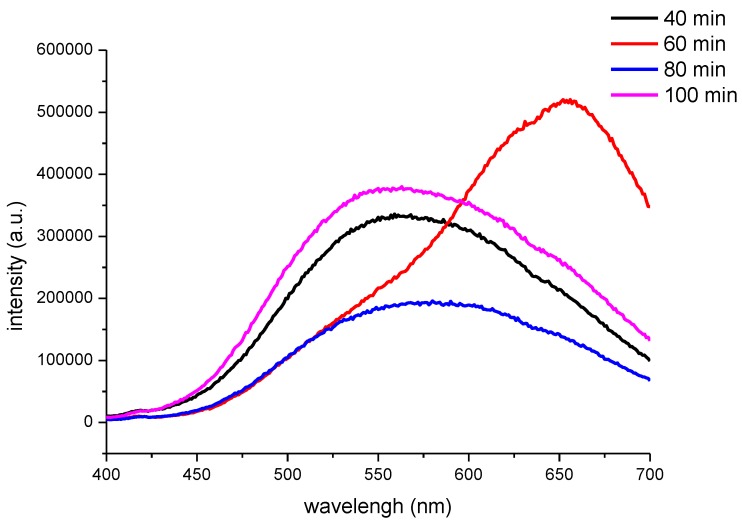
The PL spectra of the CNF/CdS nanocomposites with different maturing time (excitation wavelength 365 nm).

For these experiments, the –COOH/Cd^2+^ ratio was 1.0 and the feeding time of Na_2_S was 20 min. The effect of ultrasonic maturing time on the PL spectrum of the CNF/CdS nanocomposite was investigated. The maturing time in the ultrasonic setup was 40 min, 60 min, 80 min, and 100 min, respectively. The results are shown in Figure 6 and Figure 7.

Figure 6 illustrates the fluorescence image of the CNF/CdS nanocomposite obtained under different ultrasonic maturing time conditions (λ = 365 nm UV light). It could be seen that the CNF/CdS nanocomposite showed a red color under the excitation of the UV light only when the maturing time was 60 min, while the composite showed a yellow-green color when the ultrasonic maturing time was 40 min, 80 min, or 100 min.

Figure 7 shows the PL spectra of the CNF/CdS nanocomposite with different maturing times. The excitation wavelength used was 365 nm. It could be seen that the PL peak of the CNF/CdS nanocomposite was at 560 nm when the maturing time was 40 min. When the maturing time was 60 min, a strong emission peak at 655 nm could be observed in the PL spectrum. When the maturing time was further prolonged to 80 min and 100 min, the emission peaks showed blueshifts at 580 nm and 546 nm, respectively. The wavelength at 655 nm was red light, which is consistent with the result of the fluorescence image with the UV light (Figure 6). When the maturing time was 40 min, 80 min, or 100 min, however, the emission peak in the PL spectrum showed an obvious blueshift phenomenon. The emission wavelength was in the range of 546–580 nm, which belonged to yellow-green light. This was also consistent with the results of the fluorescence imaging.

**Figure 8:**
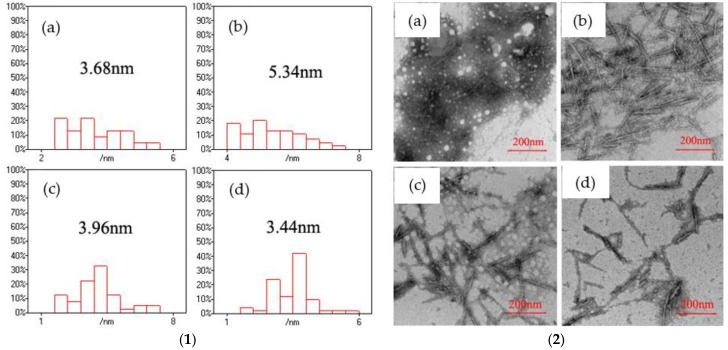
(**1**) The size distributions of the CdS nanoparticles with different maturing time; and (**2**) the TEM images of CNF/CdS nanocomposites with different maturing time; (**a**) 40 min; (**b**) 60 min; (**c**) 80 min; and (**d**) 100 min (–COOH/Cd^2+^ ratio 1.0, Na_2_S feeding time 20 min).

The morphology of CNF/CdS nanocomposite was observed by TEM. The size of the CdS particles on the CNFs was calculated using the Nano Measurer software. The results are shown in Figure 8. It could be seen that the average sizes of the CdS nanoparticles in the CNF/CdS nanocomposite were 3.68 nm, 5.34 nm, 3.96 nm, and 3.44 nm when the maturing times were 40 min, 60 min, 80 min, and 100 min, respectively. Therefore, the average size of the CdS nanoparticles was at a maximum when the maturing time in the ultrasonic setup was 60 min. This was because the cavitation of the ultrasonic treatment had two effects on the CdS nanoparticles, i.e., induced crystallization and shearing. When the maturing time in the ultrasonic equipment was short (40 min), the growth of CdS grains was incomplete, so the size of the obtained CdS nanoparticles was relatively small. When the maturing time was 60 min, the CdS grains grew completely and the average particle size reached the maximum value. If the maturing time was further prolonged, the CdS nanoparticles were broken due to the continuous shearing effect of the ultrasonic treatment. As a result, the average particle size of CdS decreased. The studies of Dai et al. [17] showed the particle size of CdS nanoparticles with 60 mM 3-mercaptopropionic acid (MPA) as a function of incubation time. It was observed that the growth of the CdS particles rapidly increased at the initial stage and gradually slowed down, indicating the MPA inhibition on CdS particle growth. In this study, the size of CdS particles was related to the maturing time in the ultrasonic setup. However, the CdS nanoparticles had smaller sizes due to shearing effect of the ultrasonic when the maturing time was too long. The results were consistent with the PL spectra. In other words, the CNF/CdS nanocomposite showed only red when the maturing time was 60 min. If the maturing time was 40 min or prolonged to 80 min and 100 min, the size of the resultant CdS nanoparticles decreased significantly, and the emission peak of the PL spectra showed an obvious blueshift phenomenon. Therefore, during the preparation of the CNF/CdS nanocomposite material, the size of the CdS nanoparticles could be adjusted by controlling the maturing time for the ultrasonic maturing. Accordingly, the PL performance could be adjusted and its variation was very significant.

### 2.4. Effect of CdS Particle Size on CNF/CdS Nanocomposite PL Performance

**Figure 9:**
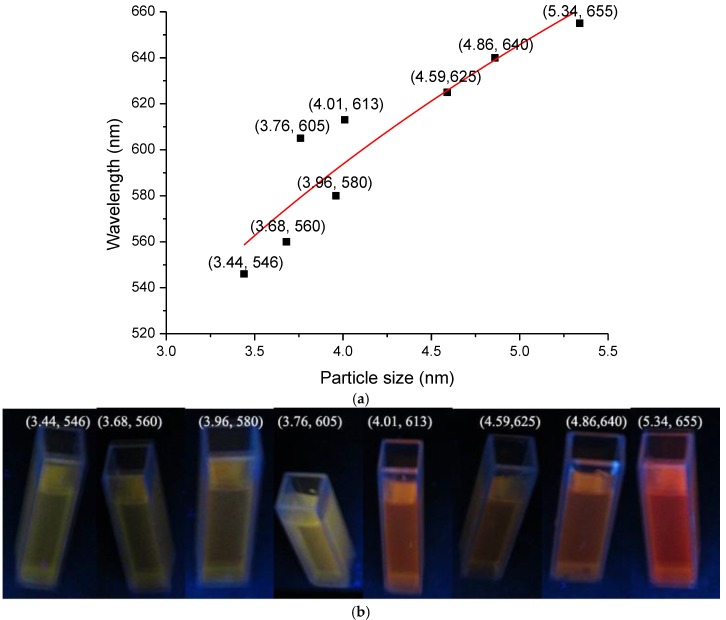
(**a**) The effect of CdS quantum dot size on the PL performance; and (**b**) the fluorescence images of the CNF/CdS nanocomposites under different CdS quantum dot sizes.

The effect of CdS quantum dot size on the PL performance was analyzed according to the size of the CdS particles (*S*) and the fluorescence emission wavelength (λ) of the CNF/CdS nanocomposite material based on the above analysis data. The results are shown in Figure 9. When the size of the CdS particles increased from 3.44 nm to 5.34 nm, the fluorescence emission wavelength of the CNF/CdS nanocomposite gradually redshifted from 546 nm to 655 nm, and their fluorescence color changed from green, to yellow, to red. In addition, the larger the CdS size was, the more significant the redshift was. This was in agreement with the quantum confinement effect [22,23,24]. Based on the data in Figure 9, the size of the CdS particles (*S*) and the fluorescence emission wavelength (λ) of the composite material were fitted. The relation between λ and *S* could be expressed as follows (Equation (2)). The correlation coefficient was 0.850.

λ = 271 + 233 ln(*S*), (2)

The results indicated that the PL performance of the CNF/CdS nanocomposite was decided by the size of CdS quantum dot. The size adjustable CdS quantum dot could be prepared by controlling the –COOH/Cd^2+^ ratio, the feeding time of Na_2_S and the maturing time in ultrasonic. Therefore, a PL performance tunable CNF/CdS nanocomposite could be obtained.

## 3. Materials and Methods

### 3.1. Materials

The bleached Eucalyptus kraft pulp was donated by Aracruz Celulose (Espirito Santo, Brazil) with a α-cellulose content of 86.8% and the degree of polymerization 1030. The other materials used in this study and their respective sources are as follows:

- CdCl_2_: Tianjin Fu Chen Chemical Reagents Factory, Tianjin, China, AR;
- Na_2_S: Guangzhou Chemical Reagent Factory, Guangzhou, China, AR;
- TEMPO: Alfa Aesar China, Shanghai, China;
- NaClO: 10%, Tianjin Fuyu Fine Chemical Co., Ltd., Tianjin, China;
- NaBr: Tianjin Kemiou Chemical Reagent Co., Ltd., Tianjin, China, AR.

### 3.2. Methods

#### 3.2.1. Preparation of CNFs

The oxidized celluloses were prepared with bleached eucalyptus kraft pulp by oxidization in a TEMPO/NaBr/NaClO alkaline medium. The detailed preparation process is described in [25]. The oxidized celluloses with a carboxyl content of 1.63 mmol/g were dispersed in deionized water. The dispersion concentration was 0.3% (*w/w*). Continuous ultrasonic wave equipment (Guangzhou Newpower Ultrasonic Electronic Equipment Co. Ltd., Guangzhou, China) was used with a working period of 10 s and a pause period of 5 s. The CNF water suspension was obtained for future use.

#### 3.2.2. Preparation of CNF/CdS Nanocomposite

A CNF water suspension (100 g) (carboxyl content of 1.63 mmol/g, concentration of 0.3% (*w/w*)) was sampled. The amount of Cd^2+^ was calculated according to the –COOH/Cd^2+^ ratio. A CdCl_2_ solution (50 mL) of a certain concentration was added dropwise into the CNF water suspension under mechanical stirring in a water bath at 20 °C. The adsorption time of the CdCl_2_ solution was controlled for 20 min. After adding the CdCl_2_ solution, the suspension was further stirred for 1 h. After that, the suspension was rapidly transferred into an ultrasonic instrument. Then, the suspension was diluted to 50 mL, followed by the addition of the same amount of Na_2_S solution. The maturing time in the ultrasonic instrument was 40–100 min. The operation mode of continuous ultrasonic wave equipment was again a working time of 10 s and a pause of 5 s. The nanocomposite materials obtained under different reaction conditions were dialyzed for one week with deionized water and stored in the dark for future use.

#### 3.2.3. TEM Analysis of CNF/CdS Nanocomposite

The morphology of CNF/CdS nanocomposite material was analyzed by a TEM (H-7650 HITACHI Corporation, Tokyo, Japan). Between 80 and 100 CdS particles in the TEM image were selected for statistical analysis using the Nano Measurer software (Microsoft Corporation, Redmond, WA, USA) to obtain the nanoparticle size distribution.

#### 3.2.4. PL Spectrum of CNF/CdS Nanocomposite

The PL spectrum of the CNF/CdS nanocomposite was measured by a fluorescence spectrophotometer (F-7000 HITACHI High-Technologies Corporation, Tokyo, Japan). The excitation wavelength was 365 nm.

## 4. Conclusions

The CNF/CdS nanocomposites with tunable photoluminescence were successfully prepared by an in situ uniformly compositing the quantized CdS nanoparticles onto CNFs. The CdS nanoparticles size and the emission wavelength of the CNF/CdS nanocomposite could be controlled by the following three ways. Firstly, by controlling the –COOH/Cd^2+^ ratio, the size of CdS nanoparticle could be changed from 4.01 nm to 5.34 nm, and the emission wavelength of the CNF/CdS nanocomposite varied from 613 nm to 655 nm. Secondly, by prolonging the feeding time of Na_2_S from 10 min, 30 min to 40 min, the size of the CdS particles changed from 3.76 nm, to 4.86 nm, to 4.59 nm. The PL peak of the CNF/CdS nanocomposite showed a red shift, changing from 604 nm to 640 nm, then blueshifted to 625 nm. Thirdly, by changing the ultrasonic maturing times from 40 min to 100 min, the CdS nanoparticles size could be changed from 3.44 nm to 5.34 nm, and the fluorescence color of the CNF/CdS nanocomposite changed from green to red with the emission wavelength varying from 546 nm to 655 nm.

Therefore, the size of the CdS nanoparticles could be controlled from 3.44 nm to 5.34 nm by adjusting the –COOH/Cd^2+^ ratio, the feeding time of Na_2_S and the ultrasonic maturing time. As a result, the PL performance of the CNF/CdS nanocomposite could be adjusted gradually from 546 nm to 655 nm, realizing the PL tunable effect. The maturing time in ultrasonic conditions showed the most significant influence on the adjustment of PL performance. This CNF/CdS nanocomposite with a tunable PL wavelength has wide potential applications in biomarkers, anti-forgery ink, and photoelectric devices.

## Abbreviations

The following abbreviations are used in this manuscript:

CNFs	Cellulose nanofibrils
TEM	Transmission Electron Microscope
PL	Photoluminescence
TEMPO	2,2,6,6-tetramethylpiperidine-1-oxyradical
MPA	3-mercaptopropionic acid
